# Visual Status in a Portuguese Population with Intellectual Disability

**DOI:** 10.3390/ijerph17217715

**Published:** 2020-10-22

**Authors:** Pedro Serra, Regina Costa, Nuno Almeida, António Baptista

**Affiliations:** 1 Instituto Superior de Educação e Ciências, Alameda das Linhas de Torres, 1750-142 Lisboa, Portugal; 2 Novas Olimpiadas Especiais—Special Olympics Portugal, Rua Sítio do Casalinho da Ajuda, 1300-536 Lisboa, Portugal; reginapeyroteo06@gmail.com; 3 Opening Eyes Portugal—Special Olympics Portugal, Rua Sítio do Casalinho da Ajuda, 1300-536 Lisboa, Portugal; miguel_opto@hotmail.com; 4 Centre of Physics, Campus Gualtar, School of Sciences, University of Minho, 4710-057 Braga, Portugal; abaptista@fisica.uminho.pt

**Keywords:** intellectual disability, ocular health, vision, eye care, vision screening, sports vision

## Abstract

Background: Neurosensory deprivation associated with vision is a well-known fact in people with intellectual disability (ID). This work aims to report the visual status of a population with ID in Portugal. Methods: A vision screening protocol was conducted during two Special Olympics events. The vision protocol included personal medical history, ocular health evaluation, and clinical measures, such as visual acuity (VA), binocular vision, colour vision, refractive error, and intraocular pressure. This protocol was administered to 134 subjects. Results: Half of the subjects reported that they had never attended or they did not remember having attended a previous eye exam. Additionally, 10% of them had not attended an eye exam in the immediate past three years. Half the subjects failed the VA test and 13% presented moderate Visual Impairment (VI) (VA worse than 0.5 logMAR in the best eye). Manifest ocular deviation was found in 25% of the subjects and the most common ocular health dysfunction conditions were conjunctiva hyperaemia, meibomian gland dysfunction, and lens anomalies. Refractive error correction allowed a reduction in the level of moderate VI to 3.7%. Conclusions: The population analysed showed a poor eye care attendance rate and vision-related conditions are in agreement with previous reports. The development of national strategies to promote the awareness for routine eye care in people with ID and improving accessibility to eye care services may mitigate many of the most prevalent conditions encountered.

## 1. Introduction

Intellectual disability (ID) is a multifaceted condition affecting all societies in the world. In Europe, the prevalence of ID has been estimated to be approximately 1% [1] and the Portuguese prevalence has been reported to be 0.84% [2]. People with ID are often considered as a group of the population requiring increased support, due to the higher probability of having a poor economic condition [3], having a lower education level [4], and being exposed to social exclusion [5]. It is also known that people presenting ID have poorer health status, higher mortality, and less involvement in health promotion activities [6]. This vulnerable part of society sees their condition even more aggravated when national health and social support services lack dedicated mechanisms to deal with their special needs [7]. 

One of the health issues affecting people with ID which interferes with their cognitive development, mobility independence, acquisition of information, and communication is the presence of neurosensorial problems such as vision [8] and hearing [9] impairment. Vision is a fundamental sensory mechanism because it allows humans to operate, navigate, and learn from the environment. A poor visual status influences the amount of information acquired, hampers independence, and represents an additional barrier to a person with ID [10]. Vision problems tend to be more prevalent in people with ID across all age ranges and increases with the severity of the condition [11]. Akinci et al. [12] reported in a population of children attending an Ophthalmologic unit that 76% of children with ID presented some type of ocular or refractive abnormality, contrasting with 42% of the children without ID. In adults, an extensive cross-sectional survey conducted in the Netherlands reported a prevalence of visual impairment ranging from 2.2% in young adults with mild ID and absence of Down’s syndrome to 66.7% in older adults with profound ID and Down’s syndrome [13]. Among the most prevalent vision problems were refractive errors, manifest ocular deviations, and lens opacities [14]. The most prevalent untreatable vision disorders were cerebral visual impairment and macular degeneration [14]. The longer life expectancy observed in the general population also seems to aggravate the interaction between ID and vision-related problems. Evenhius et al. [15] reported age as an increased risk factor for visual impairment and blindness, with lens opacities and ocular hypertension being among the most common causes of vision problems associated with age [14]. 

Vision impairment in ID has been the topic of study in various countries, which has led to the development of initiatives such as the Opening Eyes Program [16] or the more recent SeeAbility initiative [17]. These projects promote more awareness regarding this problem as well as national mechanisms specialised in dealing with eye care in people with ID [18]. A review of the literature has shown no evidence of data regarding vision health in ID in the Portuguese population. Although it can be argued that many conditions may show similar prevalence to studies carried out in other countries, the differences in the social spectrum and in National Health Services justify studies to characterise the Portuguese reality. This also may contribute to understanding the transnational dimension of this problem.

During the last decade, the Special Olympics, through their Health Athlete Program, has conducted a series of events, in Portugal, promoting the health status of people with ID. Opening Eyes is a branch of the health program, which deals with vision-related health status. This program comprises a vision screening followed by an intervention protocol, allowing the characterisation of the population screened, the resolution of refractive problems, or referral of non-refractive conditions. This study aims to report data on the ocular conditions found in a group of Portuguese adults with ID, using a standardised screening protocol. From a practical perspective, the data presented here may serve as a baseline for the development of larger studies to better characterise the vision-health status in the population with ID and should raise the awareness for the need of implementing health policies dedicated to assisting people with ID.

## 2. Materials and Methods 

The data presented in this retrospective cross-sectional study were collected during two consecutive Special Olympics Lions Club International Opening Eyes (SOLCIOE) vision screenings. The events took place in Portugal in 2009 and 2012. On both occasions, the vision screening was conducted by a group of 23 volunteers, comprising 15 optometrists, three ophthalmic technicians, and five lay volunteers. Before the events, all volunteers attended an informative session where they became familiarised with the SOLCIOE protocol. At the 2009 event, an international group of athletes was screened, but for this study, only data from the Portuguese national athletes were analysed. The conditions to take part in the visual screening were being Special Olympics participants, hereafter referred to as athletes, and having signed informed consent to participate in the Opening Eyes programme, by their parents, coaches, or carers. For research purposes, the authors were given access to the data by Special Olympics Portugal and the study received ethical approval from the Ethics Committee of Instituto Superior de Educação e Ciências de Lisboa.

**Figure 1:**
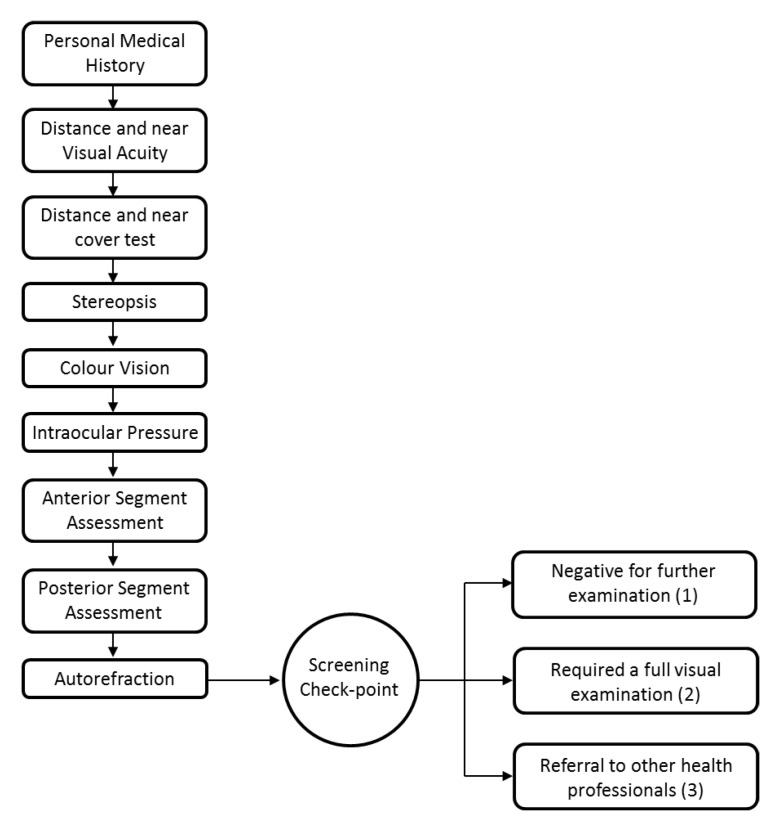
Special Olympics Lions Club International Opening Eyes (SOLCIOE) vision screenings workflow.

The screening was carried out over a series of working stations and these included (Figure 1): personal medical history, distance and near visual acuity (VA), distance and near cover test, stereopsis, colour vision, intraocular pressure (IOP), anterior segment (slit-lamp biomicroscopy) and posterior segment (direct ophthalmoscopy) eye examination, and autorefraction. A specimen of the record sheet was provided by Woodhouse et al. [19]. The tests were marked as negative/positive, i.e., pass/fail, and the decision for full refraction or referral was taken by a senior optometrist properly accredited by the Special Olympics International committee. Personal medical history was obtained and noted using multiple choice questions and presenting refraction measured with a lensometer. Distance (3.0 m) and near (0.40 m) VA were measured using LEA symbol charts which were designed so that beyond the recognition threshold, the symbols appear to be circles [20]. The charts have a precision similar to the Bailey–Lovie Chart, but better VA [21]. LEA charts use four symbols arranged in lines of five of these symbols each; the lines have a logarithmic progression in VA. Visual acuity was measured with presenting ophthalmic correction (spectacle wearers), otherwise uncorrected (non-spectacle wearers). The failing criterion was defined as monocular VA poorer than 0.3 logMAR (lower numbers represent better VA). The cover test was performed at distance and near to determine the presence, direction, and magnitude of manifest ocular deviation (tropia) or latent deviation (phoria). The criterion considered for further examination in the full refraction station was the presence of manifest ocular deviation. The Random Dot E stereotest (RDE, Stereo Optical Company, Inc, Chicago, United States of America) was used to test stereopsis at 0.50 or 0.40 m corresponding to 504 and 630 seconds-of-arc, respectively [22]. It uses a two-alternative forced-choice methodology with a side-by-side black target and a stereo plate with the letter E. The cards are presented six times and the passing criterion consisted of identifying the location of the correct plate at least five times. The RDE has been found to be effective in detecting children in need for further evaluation [23,24]. Stereoacuities finer than 504 seconds-of-arc were not screened. Colour vision was assessed using Colour Vision Testing Made Easy (CVTME, Waggoner Diagnostics, Rogers, AR, USA), a pseudoisochromatic colour plate test, which uses the identification of shapes to detect red–green colour deficits [25]. Subjects had to locate a circle in a set of nine plates; if less than eight plates were identified, the same group of plates was presented a second time and if they failed again, according to the same criterion, they were positive in the CVTME. Intraocular pressure was measured with a rebound portable tonometer (Icare^®^ TAO1i, Vantaa, Finland) and the reported value was the mean of three measurements. The Icare^®^ measures slightly higher IOP values (about 2.0 mmHg) than the Goldman tonometer (considered the gold-standard measurement device), but both techniques have good agreement [26]. A positive test was given by an IOP >21 mmHg [27].

Anterior and posterior pole assessments were performed using slit-lamp biomicroscopy and direct ophthalmoscopy. Autorefraction was performed using a handheld autorefractor (Retinomax K-plus, Nikon Corporation, Tokyo, Japan). In children, the Retinomax has been shown to have similar reproducibility (0.43D) comparing to retinoscopy, autorefraction, and subjective refraction [28]. In the adult population, the Retinomax spherical component readings are more myopic than subjective refractions [29]. The screening protocol took on average 40 to 60 min for each athlete.

After the screening process, the athlete was (1) negative for further examination if he/she passed in all stations according to the defined criteria, (2) required to attend a full visual examination (positive in the screening test) if one or more of the tests in the screening battery had a fail result, (3) referred to other health professionals in case of ocular pathology or when the use of diagnostic drugs was required. Full visual examination was carried out in the screening site and comprised static retinoscopy, subjective sphero-cylinder refinement, best corrected monocular distance visual acuity test, and cover test (Figure 1).

### Data Presentation and Analysis

The prevalence of the different visual conditions measured are presented using a similar approach to previous reports that used the same protocol [19]. Refractive error was transformed from the spherical-cylinder form to the spherical equivalent (SE = Sphere component + cylinder component/2) and cylinder components (J_0_ and J_45_) form [30] for the sake of statistical analysis and comparison with previous studies. The normality of the data was investigated using Kolmogorov–Smirnov and statistical significance analysis used parametric or non-parametric approaches. Statistical analysis was performed using IBM SPSS Statistics V23.0, IBM Corporate, New York, USA. 

## 3. Results

### 3.1. Number of Athletes

At the 2009 event, 51 Portuguese athletes were screened and in 2012, 83 athletes took part (*n* = 134 for the two events). The athletes included in the analysis participated only in one of the two screenings (Dataset available in Supplementary Materials).

### 3.2. Demographics

The total number of athletes comprised 35 females (26.2%) and 99 males (73.8%); mean age ± standard deviation was 28.6 ± 12.1 years, ranging from 11 to 65 years. Mean age for females was 28.5 ± 12.0 years and males 28.4 ± 12.7 years.

### 3.3. Success Rate for Testing

The number of athletes completing each station in the screening protocol varied (Table 1), with 84 (84/134, 62.7%) athletes completing all nine stations, 64 (64/134, 85.2%) completing seven stations, and only 12 (12/134, 9.0%) of the athletes completing fewer than four stations. The least successful station was VA assessment.

### 3.4. Last Eye Exam and Spectacle Wear

The last eye exam and spectacle wear distributions are summarised in Table 2. Regarding last eye exam date, 72 (72/134, 53.7%) athletes reported never having attended an eye exam or not knowing whether they had; however, 12 athletes wearing spectacles (12/52, 23.1%) had noticeably attended an eye examination within the last few years. Concerning spectacle wear, at assessment time, 52 (52/134, 38.8%) athletes reported using spectacles (shown in the table as “spectacle wearers”). However, of those, eight (8/52, 15.3%) did not attend the screening with spectacles and five (5/52, 9.6%) reported only using near vision spectacles. Among the 82 non-spectacle wearers (82/134, 61.9%), almost half of those athletes (34/82, 41.5%) had never attended an eye exam.

### 3.5. Symptoms

The symptoms reported by the athletes are summarised in Table 3. The most common symptom was headaches followed by photosensitivity and difficulty in seeing at distance; double vision was the least reported symptom. The symptoms reported were combined with screening results in order to find possible associations: (1) difficulty in seeing at distance vs. failing distance VA, (2) difficulty in seeing at near vs. failing near VA, (3) difficulty in seeing vs. failing distance and near VA, and (4) headaches vs. failing distance, near, or both VA. No significant associations were found (Chi-square Test: *p* > 0.05 for all), indicating that there is a poor relationship between symptoms and measured visual status. The athletes wearing spectacles were less likely to complain about difficulty in seeing at distance and near than non-spectacle wearers (Chi-square Test: χ^2^ = 5.90, *p* = 0.015).

The presence of ametropia, defined by at least one eye with a spherical equivalent higher than ±1.00 Dioptre Spherical (DS) was associated with difficulty in seeing at distance and near (Chi-square Test: χ^2^ = 4.05, *p* = 0.04). In addition, athletes presenting an astigmatism (cylinder component) above 1.00 Dioptre Cylinder (DC) at least in one eye were more likely to complain of difficulty in seeing at distance and near (Chi-square Test: χ^2^ = 4.98, *p* = 0.03). 

### 3.6. Distance and Near Visual Acuity

**Figure 2:**
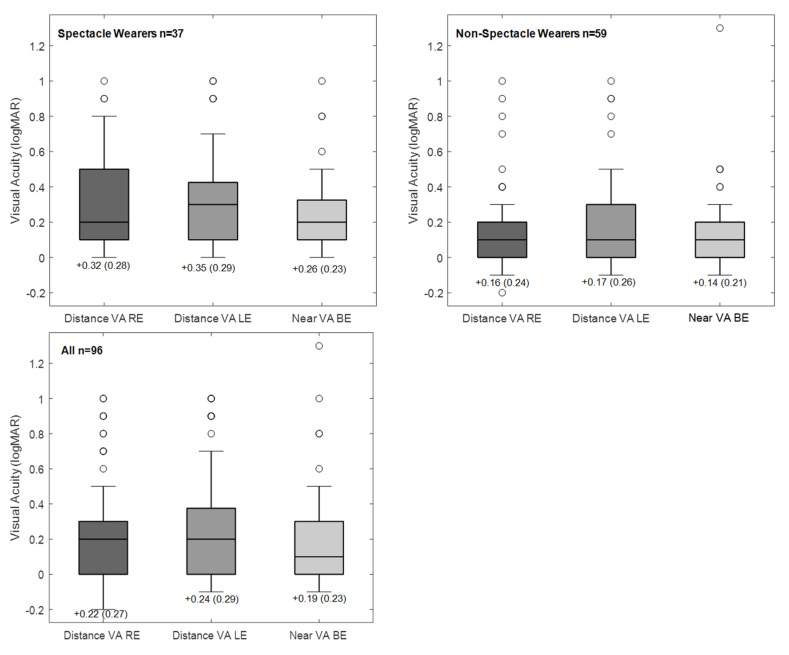
Box plots showing visual acuity (VA) distribution for the group completing distance and near VA, and for the subgroups of spectacle and non-spectacle wearers. The boxes represent the central interquartile; whiskers indicate the 95% range; the lines inside the box represent the median. Circles indicate cases outside the 95% range.

The number of athletes completing both distance (monocular) and near (binocular) VA was reduced to 96 (96/134, 71.6%). Figure 2 shows the descriptive statistics for this group and for the subgroups of spectacle (37/96, 38.5%) and non-spectacle wearers (59/96, 61.5%). Distance and near VA was successfully measured, respectively, in 101 (101/134, 75.4%) and 102 (102/134, 76.1%) athletes. VA was measured at least for one distance in 109 (109/134, 81.3%) athletes. 

For the subgroup of non-spectacle wearers, the mean VA for distance and near (Distance, RE: 0.16 ± 0.24 logMAR; LE: 0.17 ± 0.24; Near Binocular: 0.14 ± 0.21) was approximately one line better (0.1 logMAR) than the VA failing criteria (0.3 logMAR). In addition, spectacle wearers had significantly poorer vision compared (Distance, RE—0.32 ± 0.28 logMAR, LE—0.35 ± 0.29, Near Binocular—0.26 ± 0.23) to the non-spectacle wearers. Considering that, monocular distance VA was approximately 0.15 logMAR worse in the spectacle wearers group (Mann–Whitney Test: RE—U = 3.30, *p* < 0.001; LE—U = 3.66, *p* < 0.001), and the same for binocular near VA (Mann–Whitney Test: BE—U = 3.44, *p* < 0.001). 

Table 4 summarises the pass/fail criteria for VA. Forty-seven athletes (47/96, 49%) achieved distance and near VA threshold, 22 (22/96, 23%) athletes did not fulfil either distance nor near criteria, and 42 (42/96, 43.7%) had an eye with VA poorer than 0.3 logMAR. Twenty-five athletes (25/37, 67.5%) who were spectacle wearers failed the VA threshold compared to 27 (27/59, 45.8%) athletes in the non-spectacle wearers group and the difference was significant (Chi-square Test: χ^2^ = 6.58, *p* = 0.010). 

According to the 10th revision of the International Statistical Classification of Diseases and Related Health Problems classification (ICD-10-H54) [31], which considers the best eye distance VA as a threshold level to classify the level of visual impairment (VI), eighty-eight (88/101, 87.1%) athletes had no VI (better eye VA ≤ 0.5 logMAR or ≥0.3 decimal) and 13 (13/101; 12.9%) had moderate VI (better eye VA between 0.5 and 1.0 logmar (0.1 decimal)). None of the athletes presented severe VI or blindness.

### 3.7. Binocular Vision (Cover Test and Stereopsis)

The cover test at distance and near was completed in 111 (111/134, 82.8%) athletes. The majority of the athletes had no manifest ocular deviation (distance and near: 82/111, 73.9%) and manifest deviation was present in 28 (28/111, 25.2%) athletes (Table 5). One of the individuals had bilateral nystagmus. 

Stereopsis was successfully assessed in 105 (105/134, 78.4%) athletes, with 40 (40/105, 38.1%) of these failing to pass. The number of athletes completing both stereopsis and the cover test at distance was 94 (94/105, 70.1%) and stereopsis and the cover test at near was 99 (99/105, 73.9%). A statistically significant association was found among those failing stereopsis and the presence of manifest deviation identified at distance (Chi-square Test: χ^2^ = 14.5; *p* < 0.001) and near (Chi-square Test: χ^2^ = 11.67; *p* < 0.001), indicating a poorer depth perception in those athletes with manifest ocular deviation. 

### 3.8. Colour Vision

From the 117 (117/134, 87.3%) athletes completing the colour vision test, nineteen (19/117, 16.2%) failed to pass, showing a red–green defect. The sex-specific prevalence of colour vision defects was, respectively, 10.3% (3/28) and 17.8% (16/89) among women and men (Chi-square Test: χ^2^ = 0.90, *p* = 0.342). 

### 3.9. Intraocular Pressure

Intraocular pressure was measured in 103 (103/134, 77.9%) athletes in both eyes; the mean for the RE and LE was 16.0 ± 4.0 mmHg (range: 7.0 to 31.0 mmHg) and 15.8 ± 4.2 mmHg (range: 7.0 to 32.0 mmHg, respectively. The normal IOP threshold is considered as 21 mmHg; IOP > 21 mmHg was found at least in 1 eye in 11 (11/103, 10.6%) athletes. There was no correlation between IOP and athletes’ age (considering RE; Pearson Correlation: R = 0.002, *p* = 0.985).

### 3.10. Ocular Health

Anterior and posterior ocular segment examination was performed in all athletes. The prevalence of ocular conditions is summarised in Table 6. Fifty-four (54/134, 40.3%) athletes had an unremarkable ophthalmological exam. The most common eye conditions observed were: conjunctival hyperaemia (15/134, 11.2%), meibomian gland dysfunction (8/134, 6.0%), and pterygium/pinguecula (5/134, 3.7%). Regarding posterior segment examination, the most prevalent conditions were cataracts or lens opacities with 11 (11/134, 8.2%) and 5 (5/134, 3.7%) athletes being flagged with suspicion of glaucoma. 

### 3.11. Autorefraction (Refractive Error)

**Figure 3:**
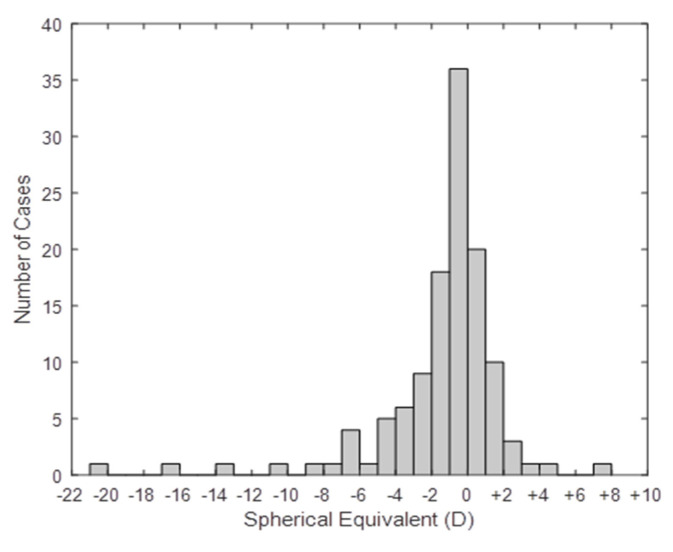
Spherical Equivalent refractive error distribution, for the present population (*n* = 122).

Refractive error analysis was assessed using the autorefraction readings. The data from 122 (122/134, 91%) athletes were analysed and the RE was selected for presentation since the inter-eye refractive error component comparisons were similar (Wilcoxon Signed Rank Test: M (Spherical Equivalent): z = −0.42 *p* = 0.672; Cylinder components: J_0_: z = −1.41 *p* = 0.158; J_45_: z = −1.01 *p* = 0.314). The spherical equivalent distribution showed a tendency towards myopia (Figure 3). Myopes of different magnitudes represented 69.7% (85/122) of the population screened, with 49 (49/122, 40.2%) having a spherical equivalent lower than −1.00 DS, while hyperopia above +1.00 DS was only observed in 16 (16/134, 13.1%) of the athletes.

The refractive error components sphere and cylinder were divided according to the magnitude of the component [19] for all athletes and comparisons were made between spectacle usage groups and gender. Low refractive errors were the main refractive status for the observed population (Table 7), 97 (97/122, 79.5%) athletes had a sphere component between ± 3.00 DS, and 81 (81/122, 66.4%) had an astigmatic component below 1.00 DC. Spherical component distribution for the spectacle wearers group failed to reach statistical significant difference from the non-spectacle group (Mann–Whitney U Test, U = 1378, *p* = 0.064), but the cylinder component differed between these two groups (Mann–Whitney U Test, U = 1060.5, *p* < 0.001). 

### 3.12. Gender

Additional to the colour vision and refractive error, no significant statistical differences between genders were found for frequency of spectacle usage, last eye examination, passing distance and near VA threshold, presence of binocular vision abnormalities, passing the stereopsis, and IOP.

### 3.13. Full Refraction and Intervention 

A full refraction examination was attempted in 69 (69/134, 51.5%) athletes for whom the final refractive correction was calculated based on static retinoscopy and/or subjective refraction. Retinoscopy was successfully performed in 68 (68/69, 98.6%) and subjective refraction in 59 (59/69, 85.5%) athletes. For those undergoing a full subjective refraction distance, VA was successfully assessed monocularly in all. Visual acuity comparison between presentation and post full refraction was possible in 49 (49/69, 71.0%) refracted athletes (21 already spectacle wearers, 28 non-spectacle wearers). For the refracted group, the mean VA presentation was 0.42 ± 0.45 logMAR and 0.44 ± 0.46 logMAR for RE and LE, respectively. On average, the full refraction improved significantly VA in both eyes by approximately two to three logMAR lines (RE: 0.28 ± 0.44 logMAR, Wilcoxon Signed Rank Test: z = 5.03 *p* < 0.001; LE: 0.27 ± 0.43 logMAR, z = 4.77 *p* < 0.001). Twenty-one (21/49, 42.8%) refracted athletes did not show any improvement in VA, 16 (16/49, 32.7%) improved between one and two VA lines, and 12 (12/49, 24.5%) improved more than three VA lines. Distance VA had a higher improvement in the athletes which were already spectacle wearers (RE: 0.36 ± 0.57 logMAR; LE: 0.34 ± 0.58 logMAR) compared to those not using spectacles at the time of the event (RE: 0.23 ± 0.36 logMAR, Wilcoxon one-sample z = 6.08 *p* < 0.001; LE: 0.24 ± 0.33 logMAR, Wilcoxon one-sample z = 4.79 *p* < 0.001).

After full refraction, 50 new prescriptions were provided to the athletes, 45 distance corrections, 5 near corrections, and 7 athletes were referred to other health professionals (ophthalmologists). According to the ICD-10 classification at the end of the program, distance VA had been assessed in 107 athletes resulting in 103 (103/107, 96.3%) athletes with no or mild VI and 4 (4/107, 3.7%) with moderate VI.

## 4. Discussion

This study reports the visual status of a group of subjects with ID that participated in the Special Olympics program. The present results concur with previous reports that analysed the same type of athletes [19,32,33]. The main findings indicate that nearly one-quarter of the population had never had an eye exam prior to the screening and approximately half of the population failed to achieve the VA threshold (VA better than 0.3 logMAR). In addition, among the most common ocular conditions were the presence of refractive error, manifest ocular deviation, lens opacities, and blepharitis. 

The SOLCIOE screening protocol could be successfully applied in a large majority of participants, as previous studies have shown [19,32,33]. The least successful station was VA assessment, a task which requires a long period of observation of geometric objects, with repetitive moments of attention to discriminate the object and the spelling out or pointing to the objects observed. Although the severity of ID was not recorded in the present study, it is likely that participants presenting more severe ID were those where VA could not be assessed. For those cases that are unresponsive to the standard test, other techniques such as the preferential looking tests could be used instead [34,35]. Other approaches that help improve the rate of response/successful testing (e.g., stereopsis and cover test) are improving the clinician’s communication skills and providing them with alternative methodologies to perform the assessment such as applying more objective techniques [36].

In this study, more than one-third (34%) of the athletes screened reported they had never attended one eye exam and an additional 10% indicated having had an eye exam more than three years ago. These figures are similar to those reported for a group of Special Olympics athletes in India [33], but are two times higher than those reported in a United Kingdom-based study [19] or in a study enrolling athletes from different European countries [32]. A study in a group of institutionalised people in Scotland reported that 80% of them had no register of a previous eye exam [37]. Several aspects may contribute towards low attendance to eye care services such as the poor awareness and knowledge of carers concerning visual problems in people with ID [38], the lack of dedicated healthcare services and trained health professionals to deal with ID [39], and difficulty in conducting an eye exam in people which often lack clear communication [18].

Regarding VA, 51% of the athletes failed to achieve the VA threshold established in the SOLCIOE protocol, indicating that half of the population screened had some degree of abnormal vision at least in one eye. This figure is close to the 41.8% found in a population of English athletes with ID [19]. Regarding the prevalence of VI (ICD-10 classification) at the beginning of the event, 13% of the athletes had moderate VI. An SO report from south India reported a prevalence two times higher than the present study, with VI ranging from mild to blind [33]. In a study enrolling European Special Olympics athletes, 11% had moderate VI [32], a similar percentage to that found in the general population with ID [11]. Refractive error higher than ±1.00 DS [19] was present in 53.3 % of the athletes. This is in agreement with previous studies where the prevalence of ametropia has been shown to vary between 30% and 50% [11]. The present findings indicate a prevalence of moderate and high myopia of 18% (myopia < −3.00DS), and an additional 2.4% of moderate and high hyperopia (hyperopia > +3.00DS). In this study, the prevalence of high hyperopia was lower than in other reports [19,32,33]. Furthermore, astigmatism is a common type of refractive error in ID; nearly 35% of the athletes had cylinders above 1.00 DC and 9% above 3.00 DC in agreement with previous studies [19,32,33]. These figures indicate a higher prevalence of astigmatism higher than 3.00 DC in a population with ID compared to people without ID [40,41]. Astigmatism seems to be a major cause of spectacle prescription, with the amount of astigmatism in the spectacle wearers group being significantly higher than in the non-spectacles group.

Refractive error correction decreased the number of athletes with moderate VI to 3.7%, indicating that uncorrected refractive error is an important cause of VI in a population with ID [19]. Nevertheless, upon refractive error correction and absence of ocular pathology, 10.5% of the athletes had some degree of mild (VA >0.3 logMAR and ≤0.5 logMAR) and moderate VI, and nearly 42.8% of those refracted did not improve VA. This can be attributed to the presence of cerebral visual impairment, caused by malfunctioning of retrochiasmatic visual pathways [42]. A study conducted in a large of population of children with ID reported cerebral visual impairment as the main cause of VI [43]. Cerebral visual impairment affects several aspects of vision such as face and object recognition, orientation, motion and spatial processing, and visual memory [42], creating particular visual deficits among the individuals. From a practical point of view, this may require individualised approaches for the assessment and treatment of the visual deficit [44]. An interesting finding regards the better VA in the non-spectacle wearers group compared to that of spectacle wearers, which agrees with a previous report in SO athletes [19]. McCullogh et al. [37] reported an association between poor VA and severity of ID; however, no relation was found between severity and refractive error. The fact that spectacle wearers present lower VA may suggest that clinicians, when presented with a patient with poor acuity, attempt to improve the visual condition with spectacles prescription, even though the visual deficit is in part associated to cortical conditions.

The prevalence of manifest ocular deviation was approximately 25%, a percentage similar to others reported in Special Olympics athletes [19,33] and within the range (4% to 69%) reported in a general population with ID [8]. Comparing to the prevalence of manifest ocular deviations found in the non-ID adult European population (1.1%) [45], people with ID are more at risk of presenting a visual deficit associated to a poor binocular condition, such as the development of amblyopia and reduced depth perception. In this cohort, nearly 28% of the athletes identified with manifest deviation at distance had a distance VA difference between both eyes higher than 0.3 logMAR, suggesting the presence of some degree of amblyopia. Additionally, depth perception (stereopsis) was reduced in individuals with manifest deviation. Considering the high prevalence of manifest ocular deviations and the fact that many of them develop during the first years of life, regular paediatric visual check-ups should be recommended in this population. 

In this study, the prevalence of colour vision defects was higher (men: 18%; women: 10%) than previously reported in people with ID (men: range 5.8 to 9.7%; women: range 0 to 1.7%) [46]. The possible reason for this higher prevalence may be attributed to the inability of some of the athletes to understand the test and to the inability of the clinician to make the test understandable. People with ID require adjustments more frequently to the test procedure or to the language/behaviour used by the clinician in order to obtain the true result than in a non-ID population. Providing clinicians with appropriate training improves their confidence to overcome more challenging situations and produce a more accurate evaluation [47]. 

Regarding IOP, the mean value was similar to that found in people without ID [48]. Van Splunder et al. [14] reported a risk of ocular hypertension in people over 50-years-old. Warburg reported a prevalence of glaucoma of 1.2% in people with moderate, severe, and profound ID [49]. For the population analysed in this study, no significant relationship was found between age and IOP; however, the combination of IOP values and optic disk head analysis flagged five (3.7%) subjects with glaucoma suspicion. Knowing that glaucoma is one of the leading causes of blindness globally [50], that its prevalence increases with age [51] and refractive error magnitude [52], and the fact that glaucoma assessment often represents a challenge to the clinician (difficulty in measuring IOP, assessing visual fields, and observing the optic disk head), this is a relevant ophthalmic condition that may often be misdiagnosed [18].

Conjunctival hyperaemia and meibomian gland dysfunction were within the most common ocular health conditions, indicating the presence of some level of ocular surface inflammation in about 17% of the athletes. Similar percentages were found in other SO events [19]. Poor lid hygiene may be pointed out as an underlying cause of inflammation; this could be mitigated by teaching the person with ID or the carer how to improve periocular hygiene. Anomalies related to crystalline lenses (cataract or lens opacities and presence of intraocular lens) were found in about 9% of the athletes. This concurs with the fact that lens anomalies are a common ocular condition at early ages in ID [14,37], making this population more prone to suffering from glare disability and reduced contrast sensitivity earlier in life. 

At the end of the screening, 50 new prescriptions and the respective new pair of spectacles were provided to the athletes, resulting in a decrease in the level of visual impairment among the group analysed. This demonstrates that a significant number of cases with visual impairment are related to uncorrected refractive error [53]. Although follow-up of these cases was not done, future research should assess the acceptance of the optical correction and determine whether visual intervention improved athletes’ quality of life.

This study has some limitations and addressing them may help improve the visual assessment of people with ID. One of them is the poor association between the athletes’ reported symptoms and visual status measurements. Although there was an association between ametropia, spectacle wearing, and reported general difficulty in seeing, it could not be specified whether the difficulties were related to distance or near vision. In addition, the presence of headaches, which was the most prevalent symptom, was not associated with any visual status parameter. Therefore, integrating the parents/institutional carer feedback would potentially improve the rationality of the personal medical history in this kind of subject. Another limitation regards the assessment and analysis of the latent deviation and the binocular vision status. About one-quarter of the athletes were identified with latent deviation, since this population has a high prevalence of manifest deviation (strabismus) and high refractive errors [54,55]; introducing the non-standard values of latent deviation as a criterion for further examination would probably help in identifying more binocular vision problems. Ideally, the criterion for further examination should rely on the presence of a decompensated latent deviation. However, performing the binocular vision tests to identify this condition may be impractical with a great majority of individuals.

## 5. Conclusions

This population of Special Olympics athletes shares a similar spectrum of ocular conditions compared to other groups of people with ID described in the literature. Despite the limited size of the population and the particular type of population that SO athletes may represent, i.e., people presenting higher ID severity or more handicapped are less likely to participate in these types of events, the findings evidence the need for increasing the frequency of attendance to eye care services [56]. To overcome this under-served situation, dedicated national health policies and initiatives need to be developed not only to deal with vision but with other aspects of health. International experience has shown that increasing the awareness of carers families to specific health conditions, providing adjustments in health services to accommodate special populations, educating clinicians, and providing economical support for purchasing treatment are effective mechanisms to mitigate this need.

## Figures and Tables

**Table 1:** Number of athletes completing the screening stations. The stations are presented according to the screening order.

Stations	Number of Athletes *n* (%)
Personal medical history	134 (100)
Distance and near visual acuity	96 (71.6)
Cover test	111 (82.8)
Stereopsis	105 (78.4)
Colour vision	117 (87.3)
Autorefraction	122 (91.0)
Intraocular pressure	103 (77.9)
Slit-lamp biomicroscopy	134 (100)
Ophthalmoscopy	134 (100)
Total number of athletes	134 (100)

**Table 2:** Number of athletes attending an eye exam within a time interval. Athletes are classified according to whether they reported having spectacles (irrespective of whether they were wearing them for screening).

Last Eye Exam	Number of Individuals *n* (%)	Number of Non-Spectacle Wearers *n* (%)	Number of Spectacle Wearers *n* (%)
<1 year	19 (14.2)	6 (7.3)	13 (25)
>3 years	14 (10.4)	9 (11.0)	5 (9.6)
1 to 3 years	29 (21.6)	7 (8.5)	22 (42.3)
Never	34 (25.4)	34 (41.5)	0 (0.0)
Not known	38 (28.4)	26 (31.7)	12 (23.1)
Total	134 (100)	82 (100)	52 (100)

**Table 3:** Prevalence of reported symptoms.

Symptoms	Number of Athletes *n* (%)
Headaches	32 (23.9)
Photosensivity	24 (17.9)
Difficulty seeing at distance	23 (17.2)
Difficulty seeing (distance and near)	12 (9.0)
Difficulty seeing at near	11 (8.2)
Double vision	8 (5.9)
Total number of athletes	134 (100)

**Table 4:** Athletes passing the VA criteria (Distance: VA better than 0.3 logMAR monocular; Near: VA better than 0.3 logMAR binocular).

	Number of athletes		
	All *n* (%)	Spectacle Wearers *n* (%)	Non-Spectacle Wearers *n* (%)
Pass distance and pass near	47 (49.0)	12 (32.4)	35 (59.3)
Pass distance and fail near	7 (7.3)	2 (5.4)	8 (13.6)
Fail distance and pass near	20 (20.8)	12 (32.4)	8 (13.6)
Fail distance and fail near	22 (22.9)	11 (29.7)	11 (18.6)
Total	96 (100)	37 (100)	59 (100)

**Table 5:** Cover test results at two testing distances.

Type of Deviation	Number of Athletes *n* (%)	
	Distance	Near
Exotropia	15 (13.5)	15 (13.5)
Esotropia	6 (5.4)	7 (6.3)
Hypertropia	7 (6.3)	6 (5.4)
Orthophoria	76 (68.5)	59 (53.2)
Exophoria	7 (6.3)	20 (18.0)
Esophoria	0 (0)	4 (3.6)
Total	111 (100)	111 (100)

**Table 6:** Abnormalities observed on anterior and posterior segment examination.

Eye Health	Number of Athletes *n* (%)
Absence of eyelids	1 (0.7)
Blepharitis	3 (2.2)
Cataract or lens opacities	11 (8.2)
Conjunctival follicles	4 (3.0)
Conjunctival hyperaemia	15 (11.2)
Conjunctivitis	2 (1.5)
Corneal opacity	1 (0.7)
Disc abnormalities	2 (1.5)
Glaucoma suspect ^†^	5 (3.7)
Intraocular lenses	2 (1.5)
Lachrymal duct obstruction	1 (0.7)
Madarosis	1 (0.7)
Meibomian gland dysfunction	8 (6.0)
Normal Exam	54 (54.5)
Pterygium/Pinguecula	5 (3.7)
Keratoconus	1(0.7)
Retina abnormalities	1 (0.7)
Total	134 (100)

^†^ Glaucoma Suspect—flagged by an IOP >21 mmHg and a cup-disc ratio suggestive of glaucomatous changes.

**Table 7:** Athletes’ classification according to ametropia.

Spherical Equivalent (DS)	Number of athletes				
	All *n* (%)	Spectacle Wearers *n* (%)	Non-Spectacle Wearers *n* (%)	Males *n* (%)	Females *n* (%)
High myopia (≤−6.00)	10 (8.2)	9 (19.1)	1 (1.3)	8 (8.8)	2 (6.5)
Moderate myopia (−3.00; −6.00)	12 (9.8)	7 (14.9)	5 (6.7)	10 (11.0)	2 (6.5)
Low myopia (−1.00; −3.00)	27 (22.1)	8 (17.0)	19 (25.3)	19 (20.9)	8 (25.8)
Emmetropia (−1.00; +1.00)	57 (46.7)	15 (31.9)	42 (56.0)	44 (48.4)	13 (41.9)
Low hyperopia (+1.00; +3.00)	13 (10.7)	5 (10.6)	8 (10.7)	7 (7.7)	6 (19.4)
Moderate hyperopia (+3.00; +6.00)	2 (1.6)	2 (4.3)	0 (0)	2 (2.2)	0 (0)
High hyperopia (>+6.00)	1 (0.8)	1 (2.1)	0 (0)	1 (1.1)	0 (0)
Total	122 (100)	47 (100)	75 (100)	91 (100)	31 (100)
		U= 1378, *p* = 0.064		U = 1387, *p* = 0.897	
**Cylinder (DC)**					
No significant cylinder (0.00; 1.00)	81 (66.4)	20 (42.6)	61 (81.3)	59 (64.8)	22 (70.7)
Low cylinder (1.00; 3.00)	30 (24.6)	19 (40.4)	11 (14.7)	24 (26.4)	6 (19.4
High cylinder (>3.00)	11 (9.0)	8 (17)	3 (4.0)	8 (8.7)	3 (9.7)
Total	122 (100)	47 (100)	75 (100)	91 (100)	31 (100)
		U = 1060.5, *p* < 0.001		U = 1414.5, *p* = 0.949	

Mann–Whitney U test.

## Supplementary Materials

The following are available online at https://www.mdpi.com/1660-4601/17/21/7715/s1, Study data set.
